# Characterization of murine polyspecific monoamine transporters

**DOI:** 10.1002/2211-5463.12183

**Published:** 2017-01-09

**Authors:** Yamato Miura, Takeo Yoshikawa, Fumito Naganuma, Tadaho Nakamura, Tomomitsu Iida, Anikó Kárpáti, Takuro Matsuzawa, Asuka Mogi, Ryuichi Harada, Kazuhiko Yanai

**Affiliations:** ^1^ Department of PharmacologyTohoku University Graduate School of MedicineSendaiMiyagiJapan; ^2^ Division of PharmacologyFaculty of MedicineTohoku Medical and Pharmaceutical UniversitySendaiMiyagiJapan

**Keywords:** monoamine transporter, polyspecific transporter, solute carrier family

## Abstract

The dysregulation of monoamine clearance in the central nervous system occurs in various neuropsychiatric disorders, and the role of polyspecific monoamine transporters in monoamine clearance is increasingly highlighted in recent studies. However, no study to date has properly characterized polyspecific monoamine transporters in the mouse brain. In the present study, we examined the kinetic properties of three mouse polyspecific monoamine transporters [organic cation transporter 2 (Oct2), Oct3, and plasma membrane monoamine transporter (Pmat)] and compared the absolute mRNA expression levels of these transporters in various brain areas. First, we evaluated the affinities of each transporter for noradrenaline, dopamine, serotonin, and histamine, and found that mouse ortholog substrate affinities were similar to those of human orthologs. Next, we performed drug inhibition assays and identified interspecies differences in the pharmacological properties of polyspecific monoamine transporters; in particular, corticosterone and decynium‐22, which are widely recognized as typical inhibitors of human OCT3, enhanced the transport activity of mouse Oct3. Finally, we quantified absolute mRNA expression levels of each transporter in various regions of the mouse brain and found that while all three transporters were ubiquitously expressed, Pmat was the most highly expressed transporter. These results provide an important foundation for future translational research investigating the roles of polyspecific monoamine transporters in neurological and neuropsychiatric disease.

**Abbreviations**
CaMKII: Ca^2+^/calmodulin‐dependent protein kinase II
OCT: organic cation transporter
PMAT: plasma membrane monoamine transporter
SNRI: serotonin‐noradrenaline reuptake inhibitor
SSRI: serotonin‐selective reuptake inhibitor
TEA: tetraethylammonium

Two distinct uptake systems are responsible for the transport of extracellular monoamine neurotransmitters in the brain. The uptake1 system facilitates the intracellular transport of extracellular neurotransmitters through high‐affinity [i.e., lower Michaelis constant (*K*_m_) value] and low‐capacity [i.e., low maximum transport velocity (*V*_max_) value] transporters with high substrate specificity. Noradrenaline uptake through the noradrenaline transporter is one example of uptake1 transport. Synaptic reuptake through the uptake1 transport system plays a central role in limiting the extent of neurotransmission, and accordingly this system is accepted as a highly important system in the brain. To this end, multiple substrate‐specific transporter inhibitors such as serotonin‐selective reuptake inhibitors (SSRIs) and serotonin–noradrenaline reuptake inhibitors (SNRIs) have been developed and implemented for clinical use 1. However, SSRIs and SNRIs fail to produce sufficient therapeutic effects in many depressive patients 2, suggesting that another monoamine transport system may be involved in monoamine clearance 3. In contrast with the uptake1 monoamine transport system, the uptake2 system transports neurotransmitters through low‐affinity, high‐capacity (high *K*_m_ value and high *V*_max_ value, respectively) transporters in a polyspecific manner; that is, these transporters interact with at least two different substrates 4. Previously, monoamine transport through the uptake2 system was considered to primarily function in peripheral organs; however, three polyspecific monoamine transporters have been identified in the brain: organic cation transporter (OCT) 2, OCT3, and plasma membrane monoamine transporter (PMAT) 5, 6, 7, 8, 9.

Human OCT2, OCT3, and PMAT transport various brain monoamines. We recently reported that human OCT3 and PMAT were responsible for monoamine uptake by human astrocytes 10. Yet, the involvement of polyspecific monoamine transporters in human neuropsychiatric disease remains unclear. Accordingly, investigations of the murine orthologs mouse (m) Oct2, mOct3, and mPmat in models of human brain diseases could improve our understanding of their relevance to human neurological disorders. However, while the human orthologs of these transporters have been extensively investigated 5, 6, the transport kinetics and pharmacological characteristics of mOct2, mOct3, and mPmat have yet to be elucidated.

Although rodent polyspecific transporters have high amino acid homology with human orthologs, they are likely to have divergent properties. This is evidenced by the fact that rat Oct1, which has 77% amino acid homology with human OCT1, is different from the human ortholog in terms of its affinity for different monoamines and its responsiveness to various drugs 11, 12. Therefore, the characterization of mOct2, mOct3, and mPmat is critical for understanding interspecies similarities and differences in monoamine transport.

In the following work, we also compared the expression levels of mOct2, mOct3, and mPmat in various brain areas. Although previous studies have reported the distributions of individual transporters 11, 13, 14, the absolute expression of each transporter has not yet been investigated. Knowledge of mOct2, mOct3, and mPmat expression in the brain is important for determining the relative contribution of each transporter to monoamine clearance *in vivo*. Thus, we quantified absolute mRNA expression for each transporter in each of 12 brain areas using the standard curve method.

## Materials and methods

### Animals

C57BL/6J male mice (8–10 weeks of age; Japan SLC, Hamamatsu, Japan) were used in this study. Mice were treated in accordance with the Principles for Care and Use of Research Animals of Tohoku University, Sendai, Japan. All animal experiments are reported in accordance with the ARRIVE guidelines 15.

### Preparations of CHO‐K1 cells stably expressing mOct2, mOct3, and mPmat

Copy cDNAs of mOct2, mOct3, and mPmat cloned from a mouse brain were inserted into pCI‐neo vectors (Promega, Madison, WI, USA) and nucleotide sequences were confirmed by the dideoxy sequencing method. CHO‐K1 cells were then transfected with either mOct2, mOct3, or mPmat vectors using Lipofectamine LTX (Life Technologies, Carlsbad, CA, USA). Stable, high‐expressing transformants were selected using G418 disulphate (Wako, Osaka, Japan).

### Uptake assays

Uptake assays were performed as described previously 16. Briefly, cells were incubated in Krebs–Ringer phosphate HEPES (KRPH) buffer containing [^3^H]‐labeled substrate (1 μCi·mL^−1^) at 37 °C for 2–60 min (for time‐dependent uptake) or 5 min (for dose‐dependent uptake and Na^+^/Cl^−^/H^+^‐dependent uptake). [^3^H]‐labeled noradrenaline, dopamine, serotonin, and histamine were purchased from PerkinElmer (Waltham, MA, USA) and [^3^H]‐1‐methyl‐4‐phenylpyridium acetate (MPP^+^) was purchased from American Radiolabelled Chemicals (St. Louis, MO, USA). Specific uptake rates in transfected cells were calculated by subtracting uptake in mock‐transfected CHO‐K1 cells.

The dependence of transporter activity on extracellular Na^+^ and/or Cl^−^ was examined by comparing MPP^+^ transport in KRPH buffer, Na^+^‐free buffer, and Cl^−^‐free buffer 10. The influence of extracellular pH on transport activity was investigated by comparing transport in pH 6.6 MES buffer, pH 7.4 HEPES buffer, and pH 8.2 HEPES buffer. Drug inhibition assays were performed using decynium‐22, imipramine hydrochloride, tetraethylammonium (TEA) chloride, cimetidine (Sigma‐Aldrich, St Louis, MO, USA), and corticosterone (Wako). The effects of protein kinase inhibitors on mOct3 activity were investigated using H‐89, RO‐32‐0432, and KN‐93 (all from Sigma‐Aldrich).

### Absolute quantitative real‐time RT‐PCR assay

mRNA expression levels of mOct2, mOct3, and mPmat were determined in total RNA isolated from 12 regions of the mouse brain; olfactory bulb, frontal cortex, cortex, striatum, posterior cortex, hippocampus, thalamus, hypothalamus, midbrain, pons, medulla, and cerebellum. Synthesized cDNA was amplified by TaqMan^®^ qPCR (Life Technologies) for absolute quantification and β‐actin was used as an internal control. Vectors containing each cDNA were used for absolute quantification, and amplification efficiencies of qPCR were nearly 100%, with standard curve *r* ^2^ values greater than 0.995. Differences between RT‐PCR reactions in the presence or absence of reverse transcriptase are shown as the results.

### Data analysis

All data are expressed as the mean ± SE. Statistical analyses and calculations of *K*_m_, *V*_max_, and concentrations producing 50% inhibition (IC_50_) were performed using prism 6 software (Graphpad, La Jolla, CA, USA). Differences were identified using Student's *t* tests and considered to be statistically significant when *P* < 0.05.

## Results

### Monoamine transport activity of mOct2, mOct3, and mPmat

**Figure 1:**
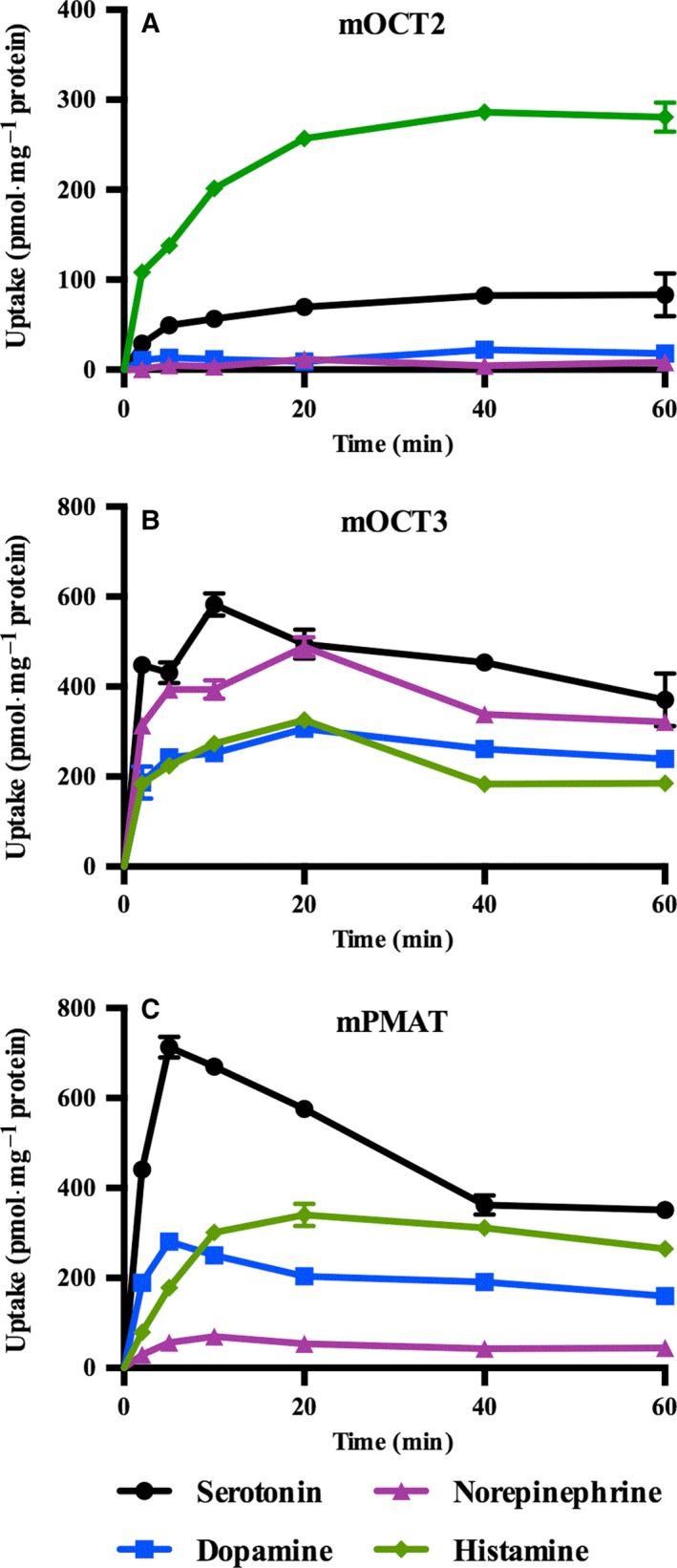
Time‐dependent uptake of serotonin, dopamine, noradrenaline, and histamine for (A) mOct2, (B) mOct3, and (C) mPmat. Time‐dependent monoamine transport assays were conducted in CHO‐K1 cells stably overexpressing mOct2, mOct3, or mPmat or control cells. Cells were incubated in the presence of 100 μm serotonin, dopamine, noradrenaline, or histamine for 2–60 min at 37 °C. Individual transport assays were performed in triplicate (three wells were analyzed for each group per trial). The results were confirmed at least two times in separate experiments using different cryopreserved cell vials. The results shown are representative of experiments performed.

We investigated time‐dependent transport of serotonin, dopamine, noradrenaline, and histamine for all three transporters. mOct2 transported serotonin and histamine in a time‐dependent manner, whereas dopamine and noradrenaline transport values were negligible (Fig. 1A). In contrast, mOct3 and mPmat transported all four neurotransmitters (Fig. 1B,C). Dose‐dependent monoamine uptake through mOct2, mOct3, and mPmat was evaluated. The resultant *K*_m_, *V*_max_, and transport efficiency (*V*_max_/*K*_m_) values are shown in Tables 1, 2, 3.

**Table 1:** Characterization of mOct2

	*K*_m_ (μm)	*V*_max_ (pmol·mg protein^−1^·min^−1^)	*V*_max_/*K*_m_ (μL·mg protein^−1^·min^−1^)
Serotonin	313 ± 68	600 ± 39	1.9
Dopamine	ND	ND	ND
Noradrenaline	ND	ND	ND
Histamine	111 ± 29	651 ± 38	5.9

ND, not determined. Individual transport assays were performed in triplicate (three wells were analyzed for each group per trial). The results were confirmed at least two times in separate experiments using different cryopreserved cell vials. The results shown are representative of experiments performed.

**Table 2:** Characterization of mOct3

	*K*_m_ (μm)	*V*_max_ (pmol·mg protein^−1^·min^−1^)	*V*_max_/*K*_m_ (μL·mg protein^−1^·min^−1^)
Serotonin	430 ± 188	3040 ± 580	7.0
Dopamine	785 ± 18	5060 ± 400	6.4
Noradrenaline	566 ± 106	10 700 ± 770	18.8
Histamine	1670 ± 290	17 200 ± 1270	10.3

Individual transport assays were performed in triplicate (three wells were analyzed for each group per trial). The results were confirmed at least two times in separate experiments using different cryopreserved cell vials. The results shown are representative of experiments performed.

**Table 3:** Characterization of mPmat

	*K*_m_ (μm)	*V*_max_ (pmol·mg protein^−1^·min^−1^)	*V*_max_/*K*_m_ (μL·mg protein^−1^·min^−1^)
Serotonin	120 ± 50	875 ± 94	7.3
Dopamine	160 ± 57	1020 ± 86	6.3
Noradrenaline	515 ± 128	1590 ± 128	3.0
Histamine	1520 ± 270	20 040 ± 980	13.2

Individual transport assays were performed in triplicate (three wells were analyzed for each group per trial). The results were confirmed at least two times in separate experiments using different cryopreserved cell vials. The results shown are representative of experiments performed.

### Effects of extracellular condition on mOct2, mOct3, and mPmat transport activity

**Figure 2:**
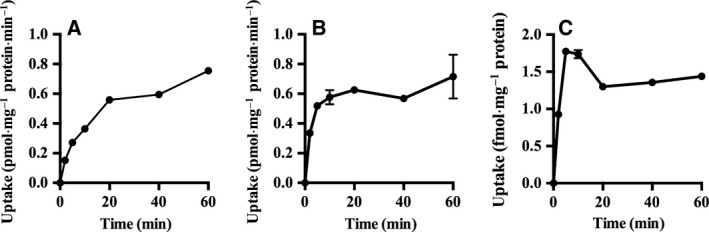
Time‐dependent transport of MPP^+^ through mOct2 (A), mOct3 (B), and mPmat (C). CHO‐K1 cells stably overexpressing mOct2, mOct3, or mPmat time‐dependently transported MPP^+^. Stable transformants and CHO‐K1 cells were incubated in the presence of 100 nm MPP^+^ for 2–60 min at 37 °C. Specific uptake in stable transformants is presented relative to that in mock‐transfected CHO‐K1 cells; Individual transport assays were performed in triplicate (three wells were analyzed for each group per trial). The results were confirmed at least two times in separate experiments using different cryopreserved cell vials. The results shown are representative of experiments performed.

**Figure 3:**
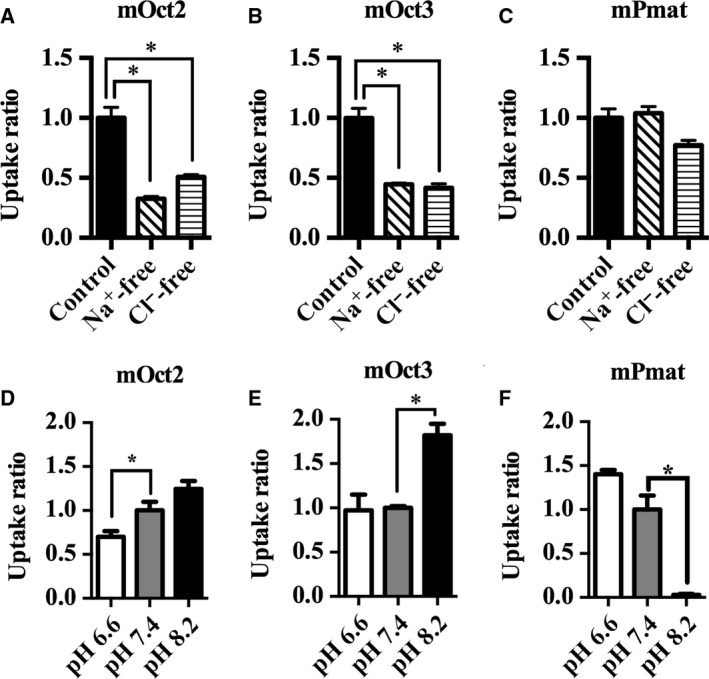
mOct2, mOct3, and mPmat activity dependence on extracellular Na^+^, Cl^−^, and pH. CHO‐K1 cells overexpressing mOct2 (A), mOct3 (B), or mPmat (C) were incubated in normal buffer, Na^+^‐free buffer, or Cl^−^‐free buffer containing 100 nm MPP^+^ for 5 min at 37 °C. MPP^+^ transport activities are expressed relative to those in normal buffer. CHO‐K1 cells overexpressing mOct2 (D), mOct3 (E), or mPmat (F) were incubated in pH‐modified buffers (pH 6.6, 7.4 or 8.2) with 100 nm MPP^+^ for 5 min at 37 °C. MPP^+^ transport activities are expressed relative to those in KRPH buffer at pH 7.4. Differences were identified using Student's *t* tests; *P < 0.05. Individual transport assays were performed in triplicate (three wells were analyzed for each group per trial). The results were confirmed at least two times in separate experiments using different cryopreserved cell vials. The results shown are representative of experiments performed.

**Figure 4:**
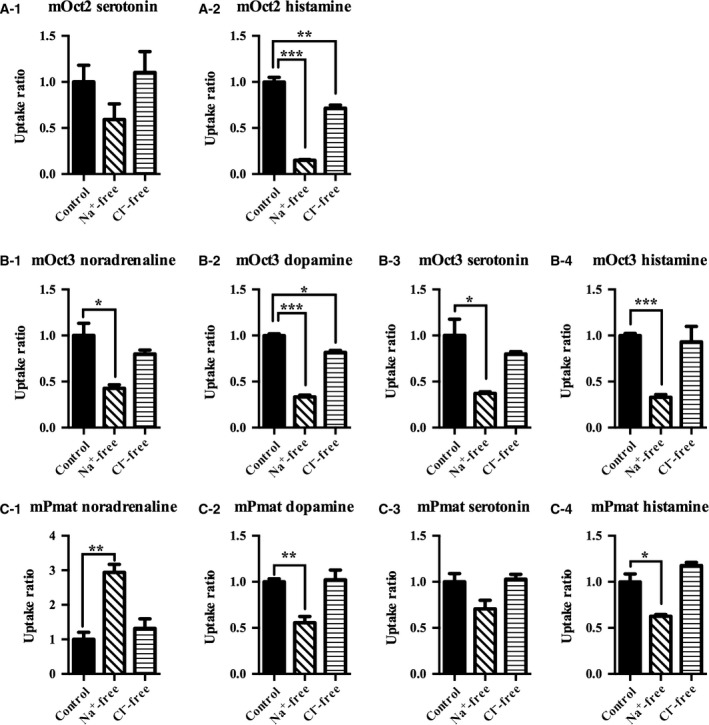
Neurotransmitter uptake activity of mOct2, mOct3, and mPmat in the presence or absence of extracellular Na^+^/Cl^−^. CHO‐K1 cells overexpressing mOct2 (A), mOct3 (B), and mPmat (C) were incubated in normal KRPH buffer, Na^+^‐free buffer, or Cl^−^‐free KRPH buffer containing 10 μm noradrenaline (A‐1, B‐1, C‐1), dopamine (B‐2, C‐2), serotonin (B‐3, C‐3), and histamine (A‐2, B‐4, C‐4) for 5 min at 37 °C. Transport activities were expressed relative to those in normal KRPH buffer. Differences were identified using Student's *t* tests; *P < 0.05, **P < 0.01, and ***P < 0.001, respectively, Individual transport assays were performed in triplicate (three wells were analyzed for each group per trial). The results were confirmed at least two times in separate experiments using different cryopreserved cell vials. The results shown are representative of experiments performed.

**Figure 5:**
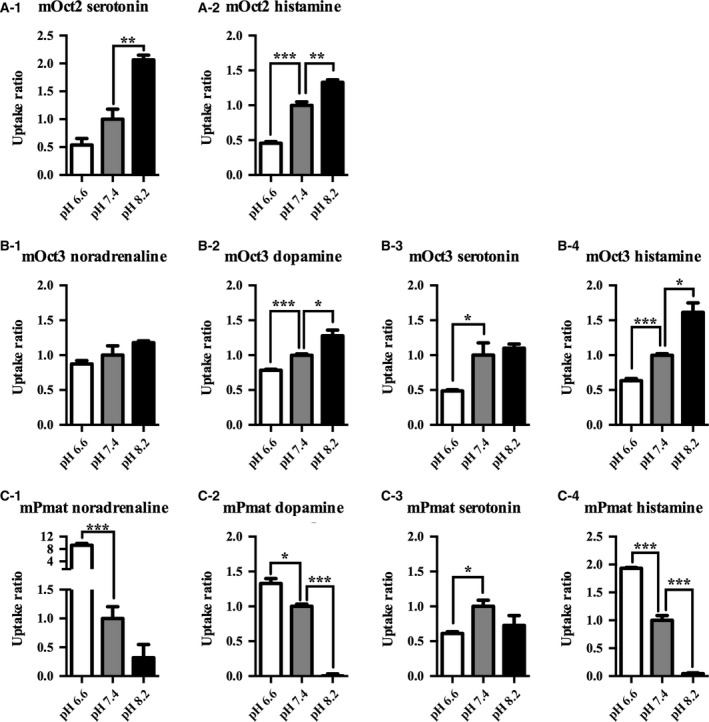
Effects of extracellular pH on neurotransmitters uptake activity of mOct2, mOct3, and mPmat. CHO‐K1 cells overexpressing mOct2 (A), mOct3 (B), and mPmat (C) were incubated in pH‐modified KRPH buffer (pH 6.6, 7.4 or 8.2) with 10 μm noradrenaline (A‐1, B‐1, C‐1), dopamine (B‐2, C‐2), serotonin (B‐3, C‐3), and histamine (A‐2, B‐4, C‐4) for 5 min at 37 °C. Transport activities are expressed relative to those in KRPH buffer at pH 7.4. Differences were identified using Student's *t* tests; *P < 0.05, **P < 0.01 and ***P < 0.001, respectively, Individual transport assays were performed in triplicate (three wells were analyzed for each group per trial). The results were confirmed at least two times in separate experiments using different cryopreserved cell vials. The results shown are representative of experiments performed.

Extracellular conditions such as Na^+^ or Cl^−^ concentrations and pH are important factors that can modulate transporter activity 17. To assess the potential influence of these factors on mOct2, mOct3, and mPmat activity, we used MPP^+^ as a substrate, which is a stable and silent compound that retains a positive charge under a variety of physiological conditions 18. The kinetics of MPP^+^ transport through mOct2, mOct3, and mPmat under physiological conditions are summarized in Fig. 2 and Table 4. In the absence of Na^+^ or Cl^−^, the transport activity of mOct2 was decreased by 70% and 50%, respectively (Fig. 3A). Moreover, in the absence of either Na^+^ or Cl^−^, the transport activity of mOct3 was decreased by 45% (Fig. 3B). Extracellular concentrations of Na^+^ and Cl^−^ had no apparent effect on mPmat activity (Fig. 3C). Next, we examined the effects of extracellular H^+^ on MPP^+^ transport. Under acidic conditions (pH 6.6), mOct2 activity was significantly decreased, whereas mPmat activity tended to increase (Fig. 3D–F). In contrast, mOct3 activity was dramatically increased under basic conditions (pH 8.2), whereas mPmat activity was almost completely abolished. We also examined neurotransmitters uptake activity of mOct2, mOct3, and mPmat in the absence of extracellular Na^+^/Cl^−^ and in pH‐modified KRPH buffer (Figs 4 and 5). In contrast to MPP^+^, neurotransmitters could be easily metabolized, protonated, and deprotonated, and might affect the intracellular signaling coupled to transporter activity. Therefore, we could not rule out the possibility that these properties of natural monoamines might affect the results.

**Table 4:** Kinetics parameters of mOct2, mOct3, and mPmat to MPP^+^

	*K*_m_ (μm)	*V*_max_ (pmol·mg protein^−1^·min^−1^)	*V*_max_/*K*_m_ (μL·mg protein^−1^·min^−1^)
mOct2	6.8 ± 0.9	197 ± 8	28.9
mOct3	116 ± 22	1890 ± 111	16.3
mPmat	32.8 ± 8.4	349 ± 20	10.6

Individual transport assays were performed in triplicate (three wells were analyzed for each group per trial). The results were confirmed at least two times in separate experiments using different cryopreserved cell vials. The results shown are representative of experiments performed.

### Pharmacological characterization of mOct2, mOct3, and mPmat

**Figure 6:**
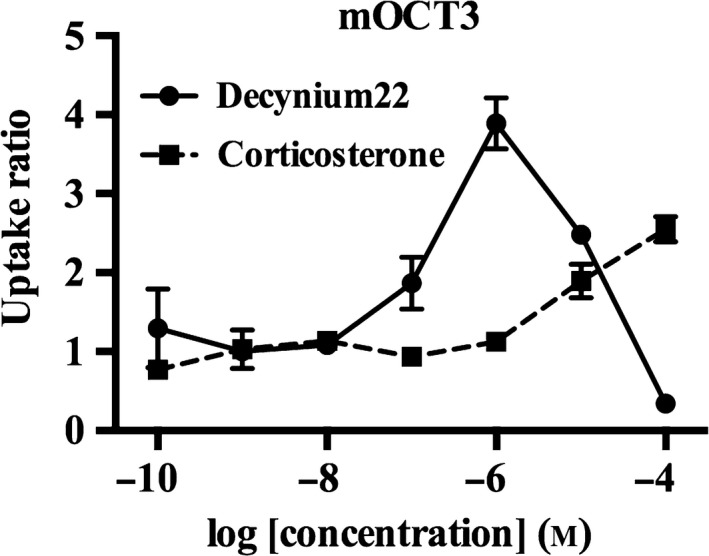
Effect of decynium‐22 and corticosterone on mOct3 transport activity. CHO‐K1 cells overexpressing mOct3 were incubated with 100 nm MPP^+^ and decynium‐22 and corticosterone for 5 min at 37 °C. MPP^+^ transport activity is expressed relative to that in untreated control cells. Individual transport assays were performed in triplicate (three wells were analyzed for each group per trial). The results were confirmed at least two times in separate experiments using different cryopreserved cell vials. The results shown are representative of experiments performed.

Drug inhibition assays were performed using imipramine, corticosterone, cimetidine, TEA, and decynium‐22, as these agents are commonly used for the characterization of cation transporters 19. All of these drugs inhibited mOct2 activity, with decynium‐22 having the lowest IC_50_ value (Table 5). Concentrations of corticosterone greater than 10 μm increased MPP^+^ transport through mOct3 (Fig. 6). Although high concentrations of decynium‐22 produced partial inhibition of mOct3, concentrations around 1 μm significantly potentiated mOct3 transport activity (Fig. 6). Imipramine, cimetidine, and TEA dose‐dependently inhibited MPP^+^ transport through mOct3. Moreover, decynium‐22, imipramine, cimetidine, and TEA, but not corticosterone, inhibited the transport activity of mPmat (Table 5).

**Table 5:** IC_50_ values of various agents for mOct2, mOct3, and mPmat activities

	mOct2	mOct3	mPmat
Cimetidine	1.34 μm	130 μm	51.8 μm
Corticosterone	8.70 μm	ND	No inhibitory effect
Decynium‐22	427 nm	ND	479 nm
Imipramine	14.2 μm	383 nm	17.2 μm
TEA	230 μm	14.8 mm	8.70 mm

ND, not determined. Individual transport assays were performed in triplicate (three wells were analyzed for each group per trial). The results were confirmed at least two times in separate experiments using different cryopreserved cell vials. The results shown are representative of experiments performed.

**Figure 7:**
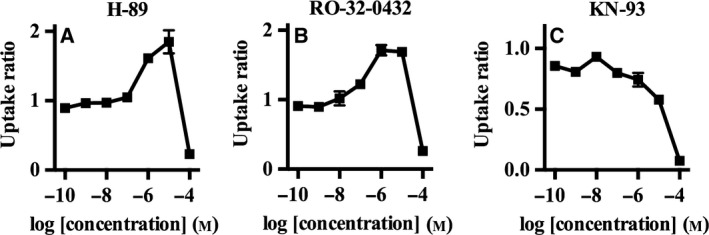
The effect of kinase inhibitors on the corticosterone‐mediated OCT3 activation. We examined the effect of corticosterone on OCT3 transport activity in the presence of 10 μm protein kinase inhibitors: protein kinase A inhibitor H‐89 (A), protein kinase C inhibitor (RO‐32‐0432) (B) and Ca^2+^/Calmodulin‐dependent kinase II inhibitor KN‐93 (C). [^3^H] MPP^+^ was used as a substrate and the incubation time was 5 min. The amount of MPP^+^ transported in to CHO‐K1 cells without corticosterone was set to 1. Individual transport assays were performed in triplicate (three wells were analyzed for each group per trial). The results were confirmed at least two times in separate experiments using different cryopreserved cell vials. The results shown are representative of experiments performed.

**Figure 8:**
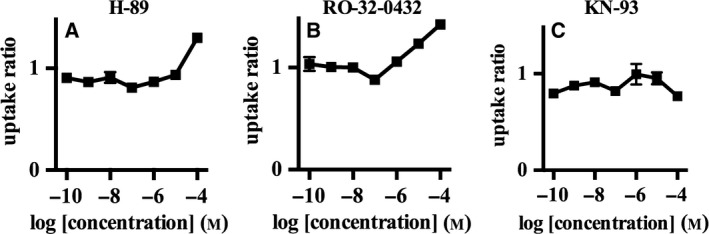
The effect of kinase inhibitors on the decynium‐22‐mediated OCT3 activation. We examined the effect of decynium‐22 on OCT3 transport activity in the presence of 10 μm protein kinase inhibitors: protein kinase A inhibitor H‐89 (A), protein kinase C inhibitor (RO‐32‐0432) (B), and Ca^2+^/Calmodulin‐dependent kinase II inhibitor KN‐93 (C). [^3^H] MPP^+^ was used as a substrate and the incubation time was 5 min. The amount of MPP^+^ transported in to CHO‐K1 cells without decynium‐22 was set to 1. Individual transport assays were performed in triplicate (three wells were analyzed for each group per trial). The results were confirmed at least two times in separate experiments using different cryopreserved cell vials. The results shown are representative of experiments performed.

We also investigated the effects of protein kinase inhibitors on corticosterone‐ and decynium‐22‐mediated enhancements in the transport activity of mOct3, since a previous study showed that structural analogs of decynium‐22 modulated the phosphorylation of rat Oct3 to enhance transport activity 20. Cotreatment with KN‐93, an inhibitor of Ca^2+^/calmodulin‐dependent protein kinase II (CaMKII), completely abolished the potentiating effects of corticosterone and decynium‐22 on mOct3 transport activity (Figs 7 and 8).

### Absolute mRNA quantification of mOct2, mOct3, and mPmat in various brain areas

**Figure 9:**
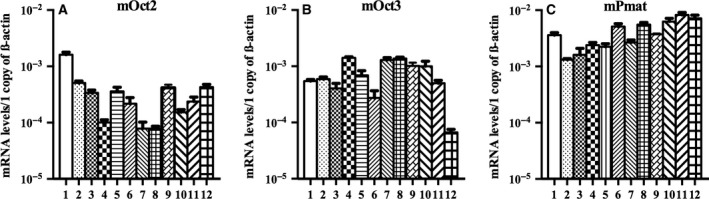
Regional monoamine transporter gene expression in mouse brains. Total RNA was isolated from the olfactory bulb (1), frontal cortex (2), cortex (3), striatum (4), posterior cortex (5), hippocampus (6), thalamus (7), hypothalamus (8), midbrain (9), pons (10), medulla (11), and cerebellum (12). mRNA expression levels for (A) mOct2, (B) mOct3, and (C) mPmat were determined in each area. Data were obtained from seven mice and are expressed as the mean ± SE.

Although the contributions of polyspecific monoamine transporters to monoamine clearance *in vivo* is partially related to their transport efficiency for various monoamines, functional expression of transporters in cellular membrane defines their impact on monoamine clearance. However, sensitive and specific antibodies against the transporters, which are required to reveal the protein expression localized to plasma membrane, are not available. Although mRNA levels do not always reflect protein levels, mRNA expression is one of the important factors to determine the amount of transporters. Therefore, the absolute expression levels of mOct2, mOct3, and mPmat were investigated using RT‐PCR. mOct2 was highly expressed in the olfactory bulb (Fig. 9A), whereas mOct3 was highly expressed in the striatum, thalamus, and hypothalamus (Fig. 9B), and mPmat expression was most notable in the medulla, cerebellum, and pons (Fig. 9C). In general, Oct2, Oct3, and Pmat were ubiquitously expressed in all brain regions; however, mPmat expression was highest relative to Oct2 and Oct3 in most brain regions.

## Discussion

This study is the first to provide a detailed characterization of polyspecific monoamine transporters in the murine brain. We demonstrated that mOct2 only transported serotonin and histamine. Moreover, MPP^+^ transport through mOct2 was dependent on extracellular Na^+^ and Cl^−^, and was enhanced in the presence of high extracellular pH. Drug inhibition assays revealed that cimetidine, decynium‐22, and imipramine decreased mOct2 transport activity at low concentrations.

Previous studies have shown that rat Oct2 and human OCT2 are selective for serotonin and histamine over dopamine and noradrenaline 11, suggesting that catecholamines are not favored substrates of the mammalian Oct2. Moreover, a previous report indicated that the activity of rat Oct2 was increased in the presence of high extracellular pH 21. These data suggest that the transport characteristics of mOct2 are similar to those of rat Oct2 and human OCT2. However, the IC_50_ values of human OCT2 for cimetidine and decynium‐22 were 171 and 13.8 μm 21, 22, whereas those of mOct2 in the present study were 1.3 μm and 427 nm, respectively, indicating an interspecies difference in the potency of pharmacological reagents.

mOct2 gene expression was detected in various brain regions including the olfactory bulb and cerebral cortex, but was generally lower than that of mOct3 and mPmat. This gene expression pattern suggests that the contribution of mOct2 to monoamine regulation in the brain is minor with respect to mOct3 and mPmat; however, we did not investigate the protein or functional expression of mOct2 or other transporters to confirm this hypothesis. Moreover, a recent study demonstrated that mOct2 regulated the tone of serotonergic neurons and controlled stress vulnerability 23. Immunoelectron staining also identified Oct2 localized to presynaptic vesicles 24. These data suggest that Oct2 may have an important role in synaptic vesicle loading and thus the regulation of neurotransmission, although electrophysiological experiments are not sufficiently performed to clarify the direct involvement of mOct2 in neurotransmission. Future studies should evaluate this possibility in order to inform the exact roles of human OCT2 in brain functions and various neurological disorders.

We found that while mOct3 transported all four monoamines assayed, higher transport efficiency values were noted for noradrenaline and histamine relative to serotonin and dopamine. Noradrenaline and histamine are also the favored substrates of human OCT3 6, indicating that mOct3 and human OCT3 have similar monoamine selectivity profiles. However, the pharmacological characteristics of these two orthologs were quite different despite a high amino acid homology (86%). Decynium‐22 is widely recognized as a common inhibitor of polyspecific monoamine transporters, and has an IC_50_ value of 0.9 μm for human OCT3 25. In contrast, decynium‐22 concentrations near 1 μm enhanced the transport activity of mOct3 in the present study. Isocyanine derivatives, which are structural analogs of decynium‐22, were also reported to increased rat Oct3‐mediated transport 20, whereas these compounds inhibited human OCT3 activity in a dose‐dependent manner in another study 8, suggesting that prominent interspecies differences exist in ortholog sensitivity to isocyanine analogs.

The activities of OCTs can be regulated by phosphorylation, and accordingly putative phosphorylation sites have been reported for polyspecific transporters 26. In the present study, we showed that decynium‐22‐mediated enhancements in mOct3 transport activity were abolished in the presence of a CaMKII inhibitor. This finding suggests that decynium‐22 promotes the phosphorylation of mOct3 by CaMKII as a mechanism to promote its activity.

Corticosterone has high inhibitory potency and efficacy for human OCT3 8. Yet, corticosterone failed to inhibit rat Oct3 in a previous study 27, and we observed paradoxical enhancement effect of corticosterone on mOct3 activity at select concentrations. Similar to decynium‐22, the effect of corticosterone on mOct3 was abolished in the presence of KN‐93, indicating that corticosterone may also influence the phosphorylation of mOct3 by CaMKII as a mechanism to enhance its transport activity. Our data highlight interspecies differences in the pharmacological properties of Oct3 that should be carefully considered in future translational research.

In the present study, mOct3 expression was prominent in the striatum, thalamus, hypothalamus, midbrain and pons. Cui *et al*. 28 reported the expression of mOct3 in striatal astrocytes and suggested a role for this expression in the uptake of dopamine released in response to methamphetamine stimulation. Gasser *et al*. 14 detected rat Oct3 expression in the dorsomedial hypothalamus and suggested a role for this expression in histamine clearance. Although electrophysiological studies are necessary to reveal the importance of Oct3 for neurotransmission, these results indicate that Oct3 is involved in the clearance of various monoamines and is likely to support a variety of brain functions.

Human PMAT has been reported to transport MPP^+^ in a manner independent of extracellular Na^+^ or Cl^−^ 7. In partial agreement, we showed that the transport of MPP^+^ and serotonin by mPmat was not dependent on extracellular Na^+^ or Cl^−^, although extracellular Na^+^ affected noradrenaline, dopamine, and histamine transport through mPmat. Decynium‐22 was previously reported to inhibit human PMAT activity with an IC_50_ value of 100 nm 7. In our study, decynium‐22 also had a potent inhibitory effect on mPmat activity. These findings suggest that mPmat and human PMAT have similar substrate preferences and pharmacological characteristics.

With regard to gene expression, mPmat was the mostly highly expressed transporter among the three polyspecific monoamine transporters investigated in this study. mPmat was most highly expressed in the medulla oblongata. Hosford *et al*. 29 reported that rat Pmat expression regulated serotonin concentrations in the nucleus tractus solitarii of the medulla. Additionally, Duan and Wang 30 showed that mPmat was involved in monoamine uptake in the choroid plexus. These results together highlight the importance of Pmat in monoaminergic neurotransmission. A human *PMAT* gene polymorphism has been reported in association with autism spectrum disorders and was posited to underlie dysfunction of the serotonergic system 31. Accordingly, the gene expression of Pmat is of significant clinical interest and warrants further research.

In the present work, we characterized the monoamine transport activities of mOct2, mOct3, and mPmat, and demonstrated several similarities and differences with respect to the human orthologs OCT2, OCT3, and PMAT. Overall, these orthologs appeared to have similar functions for monoamine clearance. Accordingly, investigating the roles of these transporters in mouse models of human disease could lead to a better understanding of disease pathophysiology and lead to the identification of novel therapeutic targets. Importantly, specific and potent inhibitors are essential for studying the pathological roles of mOct2, mOct3, and mPmat in models of human disease; the development of novel pharmacological drugs for each mouse transporter is therefore necessary.

## Conclusions

To our knowledge, this is the first study to characterize the brain distribution and pharmacological kinetics of the mouse polyspecific monoamine transporters mOct2, mOct3, and mPmat. We hope that these results will lead to improvements in the understanding of the role of the monoaminergic system and specifically the uptake2 system in various neurological and neuropsychiatric disease states.

## Author contributions

TY and KY designed the project and wrote the paper. YM, TY, FN, TN, TI, and AK acquired the data. YM, TY, TM, AM, and RH analyzed and interpreted the data.
